# Proposing candidate genes under telomeric control based on cross-species position data

**DOI:** 10.1093/nargab/lqae037

**Published:** 2024-04-25

**Authors:** Elias F Projahn, Georg Fuellen, Michael Walter, Steffen Möller

**Affiliations:** Institute for Biostatistics and Informatics in Medicine and Ageing Research, Rostock University Medical Center, Rostock, Germany; Institute for Biostatistics and Informatics in Medicine and Ageing Research, Rostock University Medical Center, Rostock, Germany; Institute for Clinical Chemistry and Laboratory Medicine, Rostock University Medical Center, Rostock, Germany; Institute for Biostatistics and Informatics in Medicine and Ageing Research, Rostock University Medical Center, Rostock, Germany; Institute for Clinical Chemistry and Laboratory Medicine, Rostock University Medical Center, Rostock, Germany

## Abstract

In this paper, we present a comprehensive computational framework aimed at suggesting genes whose transcriptional regulation is likely to be influenced by their chromosomal position. This framework provides a user-friendly web interface, enabling researchers to explore the positional properties of all human genes and their orthologs across species, with a focus on their relation to the telomeres. Our approach involves multiple scoring methods, each adjustable by users, representing different features of the genes' positional variation across species. The resulting rankings can be combined to identify candidate genes that may be subject to position effects. Furthermore, the ranking can be tailored to a specific set of reference genes. We evaluate the method within the context of TPE-OLD, a mechanism where telomeres can exert a direct influence on gene expression across considerable genomic distances, and empower researchers to delve deeper into genes of interest, analyzing their position across species and estimating their susceptibility to position effects like TPE-OLD. We also provide simple enrichment analyses of user-provided gene lists in relation to top-ranking candidate genes.

## Graphical Abstract

**Graphical Abstract:**
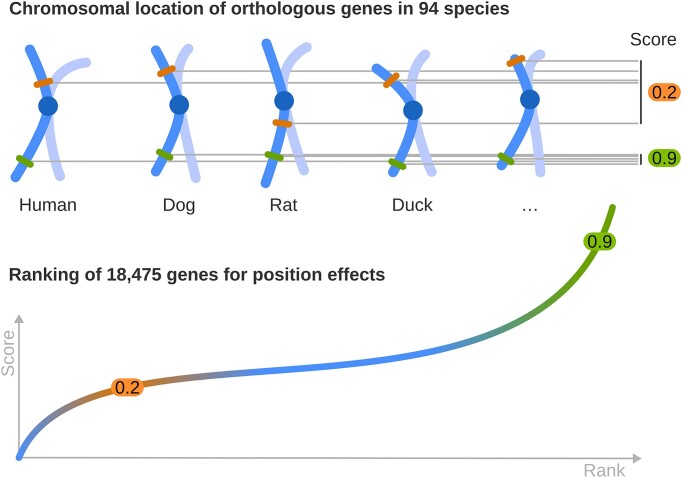

## Introduction

The structural arrangement of chromosomes plays a pivotal role in gene regulation (1), with the positioning of genes offering valuable insights into their functions (2). Notably, the terminal regions of chromosomes hold particular significance, as they are inherently mobile unless subject to specific controls that anchor them within the nuclear envelope (3). Drawing an analogy to the concept of evolutionarily conserved gene neighborhoods, which often signify shared gene functions (2), our curiosity led us to explore the possibility of identifying genes positioned near chromosomal ends across a diverse range of species. This exploration aims to suggest a potential relationship with telomeres or an anticipation of regulation by telomeric mechanisms based on the hypothesis of a functional implication of evolutionarily conserved gene positions.

Telomeres are repetitive DNA sequences that cap the ends of the linear chromosomes found in most eukaryotic cells. They protect the chromosome ends but are affected by gradual shortening during replication due to inefficient duplication of terminal DNA. Cells may have gained other benefits from the development of telomeres. Since frequent cellular divisions shorten telomeres, their lengths may serve as an input parameter to the cells’ regulome. There have been several descriptions of such effects: The Telomere Position Effect (TPE) is one phenomenon wherein telomeres are known to have an impact on the expression of proximal genes (4). Additionally, we are intrigued by a related but distinct concept known as the telomere position effect over long distances (TPE-OLD), which is believed to operate through long-distance chromatin loops that may influence genes up to 15 Mb away from the telomere. This mechanism is thought to be responsible for how telomere length can influence gene expression in human cells (5).

It is worth noting that, at the time of writing, only six genes have been experimentally verified by 3D-FISH to exhibit TPE-OLD characteristics: C1S, DSP and ISG15 (5); SORBS2 (6); TERT (7) and PPP2R2C (8). These six known TPE-OLD genes are distributed across different chromosomal arms, hinting at a chromosome-independent mechanism at play. Consequently, our investigation anticipates the presence of additional TPE-OLD genes within the unexplored chromosomal arms and in the vicinity of these earlier discoveries. This means that the methods described in this paper are one way to find more candidate genes for phenomena such as TPE-OLD. In order to make these results readily available to researchers, who wish to study these candidate genes experimentally, we present an interactive web interface accessible at https://tpe-old.uni-rostock.de.

The telomeric influence on gene regulation is a prime example of a mechanism where the chromosomal position of the gene, i.e. its distance from the telomere, is of importance to control its function in the cell. This further substantiates the hypothesis that these genes are likely to show specific patterns in their chromosomal position across species, imposing a constraint on chromosomal rearrangements. It also suggests that there may be other types of position effects, not necessarily telomere-related, that the method presented here should also be able to elucidate.

## Materials and methods

A series of five scoring methods was implemented to represent distinct patterns in the chromosomal position of human genes and their orthologs across species (Table 1). Of these methods, two describe these positional patterns directly based on the distance to the telomeres and the clustering of chromosomal positions. Additionally, three other methods can be used to base the results on a user-defined set of reference genes using statistical approaches and machine learning. The methods can be used individually to analyze different aspects of genes of interest. We also combined all methods using a weighted average and an optimization algorithm that adapts the weights to the reference gene set to refine the ranking and assess the individual methods’ contribution.

**Table 1.** Methods to rank genes based on their positions across species and results for TPE-OLD

Method	Description, *Motivation*	Mean percentile	Weight
Distance	Distance to the telomeres across species*. Control by telomeres may be more direct or more reliable when a gene is located close to the telomeres*.	29.16%	1.00
Clustering	Proportion of orthologs that have similar telomeric distances across species. *If the telomeric distance is of functional importance, i.e. if it is in some way constrained (by the chromatin arrangement), then we may expect this distance to be preserved across species*.	75.22%	0.07
Adjacency	The distribution of the telomeric distances of the gene across species is estimated. The location of the maximum density of the distribution is compared to the corresponding value for the reference genes. *This method penalizes genes that do not occur in the region typical for the reference genes, without artificially defining a fixed boundary*.	48.06%	0.70
Correlation	Spearman's rank correlation of the telomeric distances of the gene in comparison with the reference gene set is computed across species to assess if the gene is as close or distant to telomeres as the reference genes. *Not all species age replicatively* (10), *hence there may be differences in the utilization of telomeric control. Genes that are close to telomeres in species, in which the reference genes are also close, may be more likely to share regulatory mechanisms based on position*.	84.08%	0.21
Random forest	Assessment of a random forest model trained on reference gene distances. *The random forest is a machine learning method that finds its own ‘heuristics’ after seeing all reference genes’ chromosomal locations in all eligible species. It may derive patterns in positional data that have not been covered by the man-made heuristics described above*.	92.74%	0.48
Combined	Weighted average of the above scores optimized on the mean rank of the reference genes. *The aforementioned methods have different strengths, as some are more specific for the reference genes and others more sensitive. A combination of these methods is hence desirable. The weights are preset for the best performance on the reference genes, but users may want to vary these weights to interactively investigate the effect on their genes of interest*.	94.51%	-

Every method determines separate scores for each human gene considering its orthologs in the included species. The mean percentile of the known TPE-OLD genes is shown to indicate the respective methods’ performance. The rightmost column shows the optimal weights of the individual methods to achieve the highest ranks for the known TPE-OLD genes in the combined ranking, normalized on the highest individual score. See Methods for details.

### Data Retrieval and Preprocessing

The gene position data was retrieved from Ensembl, a publicly available database of genome assemblies and annotations (9, version 110, https://www.ensembl.org). We programmatically selected species based on the availability of a chromosome level assembly. In these pre-selected species, we queried for all genes that are part of a top level assembly, i.e. a chromosome, and are orthologs to human genes. The qualified genes were prefiltered based on the number of available orthologs excluding *genes* with orthologs in less than 25% of species. Subsequently, we also excluded *species* with orthologs for less than 50% of the human genes, leaving 94 species and 18,475 human genes to qualify for the analysis. In the resulting dataset, there are orthologs in more than half of the species for 86.4% of the included genes.

We used the distance to the nearest telomere in base pairs (bp) as the basis for all methods in order to normalize data for different chromosomes and chromosomal arms. Because telomeres are not included in Ensembl, the distances were approximated based on the start and end of the assembled sequence. The positions of centromeres are not available for a large proportion of species in the Ensembl database and have therefore not contributed to this method.

### Scoring methods

#### Distance and adjacency

The distance score represents the chromosomal location of each gene across species in relation to the respective closest telomere. This means that the distance score is not a direct representation of the actual chromosomal distance in the human alone. For each gene, all telomeric distances across species are used to estimate their distribution using the R function *density* from the *stats* package. The location with the maximum density is used as the distance score after normalization. This approach makes the score more robust to outliers by estimating the modal value of the distribution of gene positions across species. The distance score will be highest for genes where most of the orthologs are located far away from the telomeres. The adjacency score takes into account a set of reference genes. The distance to those reference genes (and their orthologs) substitutes the distance to telomeres for the scoring. Effectively, genes whose typical location is close to that of the reference genes, get higher scores. Reference genes are excluded from the computation of their own score.

#### Clustering

For each gene, a hierarchical clustering algorithm is applied, based on the telomere distance data for all orthologs, resulting in clusters with a predefined maximum span of 1 Mb (R functions *hclus*t and *cutree* from the *stats* package). We then quantify how many orthologs of a human gene contribute to clusters in distance data, counting the number of species in each cluster. The proportion of included species in relation to the total number of orthologs is computed separately for each cluster. The score of the largest cluster is used as the initial clustering score. To cover remaining orthologs, the scores of subsequent clusters contribute exponentially less by a factor of 0.5 per rank. This means that the largest cluster's relative size will be weighted with 100%, the second largest with 50%, the third with 25% etc. By not only considering the size of the largest cluster, the resulting score takes a broad range of clustering patterns into account while still favoring larger clusters.

#### Correlation

For each gene, its distances to the telomeres across species are compared with the respective data from all reference genes using Spearman's rank correlation coefficient (*cor* function from the R *stats* package). The median of those comparisons (masking the identity of the reference genes) is the reported score.

#### Random forest

The random forest model was implemented using the R package *ranger*. It is trained to assign high scores to genes whose positions across species match patterns found in the reference gene set. We chose a random forest instead of other machine learning methods to avoid overfitting in regard to a possibly small number of reference genes. The predictions are not made by a single model. Instead, there is one model (i.e. one random forest) for each reference gene that is trained on the remaining reference genes besides that one gene. This makes it possible to report valid results for each of the reference genes itself by basing the prediction and final score on just the model that was not trained on it. The final scores for all other genes are computed as the average of all the models’ predictions.

### Combined ranking

To generate a ranking of genes, especially for the use case of finding candidate genes based on a set of reference genes, we combined the scores of the different individual methods to compute an overall score for each gene. The individual methods’ scores are combined by computing a (positively or negatively) weighted average to either specifically highlight the influence of the individual scores or to automatically optimize the ranking based on a user-selected set of genes by minimizing their mean rank. By default, this optimization is performed based on the reference gene set. For optimizing the weights, we use the *optim* R function from the *stats* package. This results in scores that depend linearly on the scores given by the individual methods, while the amount by which each method influences the combined scores can vary.

### Visualization and presentation

Because of the exploratory nature of this research, visualization and presentation are a vital part of the methodology. We developed a large number of interactive visualizations for the input dataset, the different methods and the overall results using the *plotly* R package. To facilitate the reusability for other researchers, we developed an interactive web interface using the *shiny* package that can be used to customize the method, visualize the results and analyze genes of interest.

## Results

**Figure 1.**
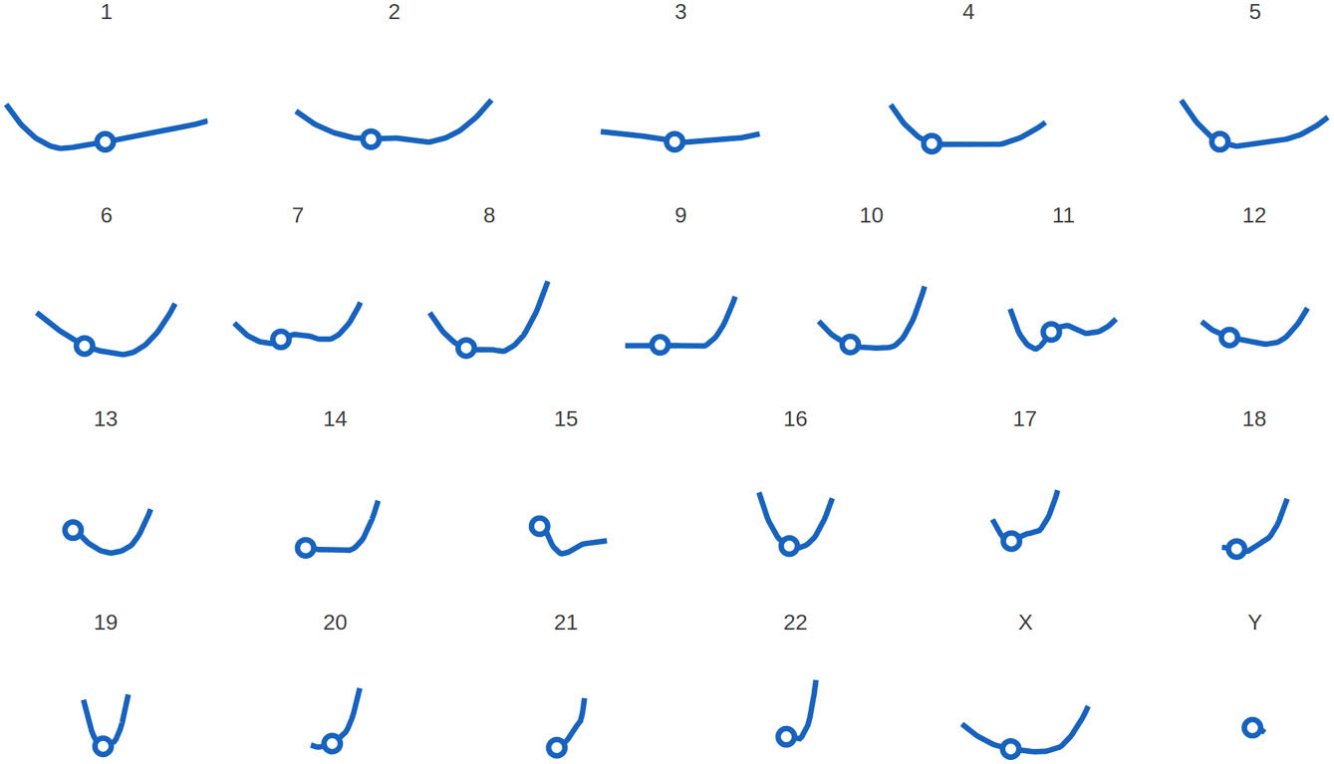
Cross-species clustering of genes by chromosomal position in the human. In a karyogram-like arrangement, the chromosomes are laid out horizontally so that the p-arm is on the left and the q-arm is on the right side. The circles indicate the respective location of the centromere. The vertical axis is consistent across all chromosomes and illustrates the scores given by the clustering analysis for genes in this chromosomal location, representing the propensity of orthologs to preserve the telomeric distance. There is a visible pattern in which the clustering score increases towards the end of the chromosomal arms. For most acrocentric chromosomes, scores are higher on the q-arm.

The methods scoring the genes’ telomeric distance and evaluating invariance of their position (‘clustering’), are the only approaches in our toolkit that operate independently of a reference gene set. Furthermore, the clustering scores are not dependent on the absolute gene position or telomeric distances. However, we observe the degree to which genes are clustering across species to be associated with their chromosomal location in the human: For most chromosomes, genes that are near the telomeres are more likely to exhibit a high clustering score. The web interface includes multiple means to visualize the individual and combined results in relation to the chromosomal position. A quick overview of all human chromosomes is provided by a simplified karyogram-like arrangement (Figure 1).

### Results with known TPE-OLD genes as reference genes

TPE-OLD serves as an example for a highly position-dependent effect on gene regulation (5). To study the method's application to TPE-OLD, we inspect the individual methods and the combined ranking upon parameter optimization using the known TPE-OLD genes as reference genes. In the resulting dataset, genes that are highly ranked alongside known TPE-OLD genes are candidates for further research on their position-dependent regulation. The full dataset with default parameters is included in the Supplement Table S1, and also made available via the web interface.

**Figure 2.**
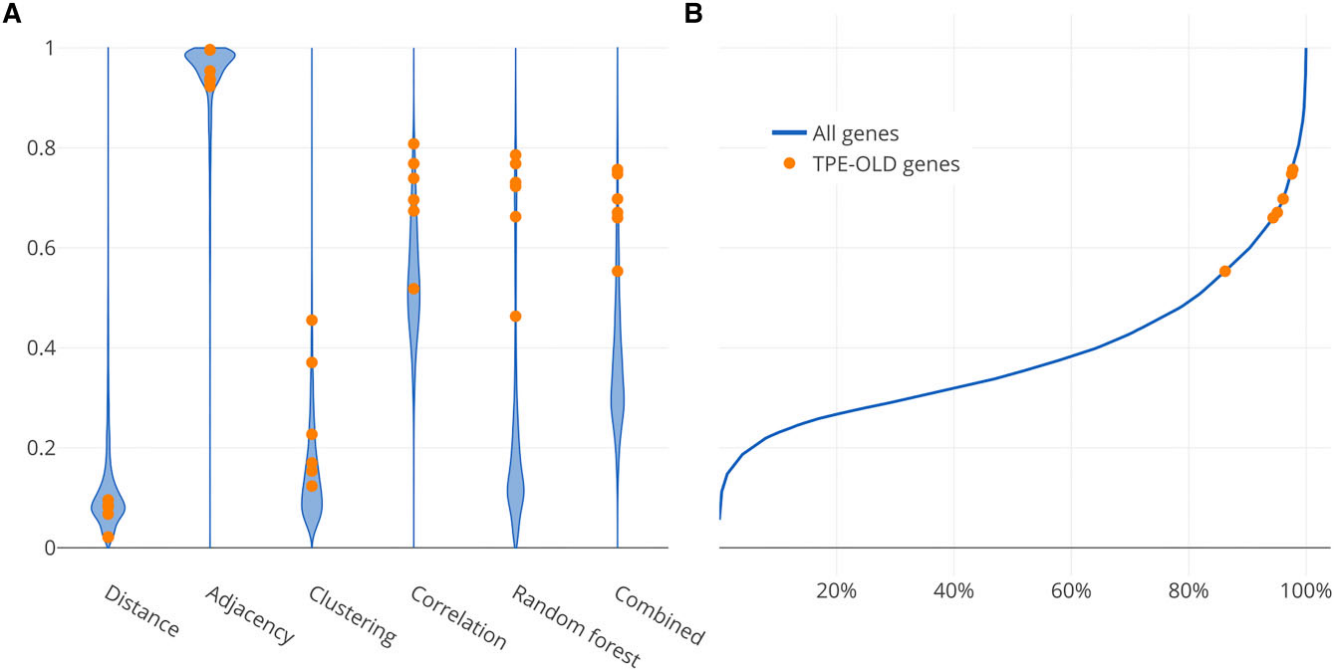
Score distribution of individual methods and results for known TPE-OLD genes. (**A**) Results for the separate methods that were applied as well as the final optimized ranking that combines all methods. The highlighted values represent the scores of the known TPE-OLD genes. Additionally, the overall distribution of scores is indicated by their density function (violin plot). For the combined ranking, the known TPE-OLD genes have particularly high scores while the majority of other genes are scored lower. (**B**) Focusing on the final combined ranking, this graph shows the distribution of scores by rank. The known TPE-OLD genes are occupying ranks within the top 15% of the combined distribution.

The separate methods have different distributions of scores and effect sizes for separating known TPE-OLD genes from other genes (Table 1, Figure 2A). Some methods, like the adjacency score, give relatively high scores to all genes, filtering out some unlikely candidates. Other scores, like the random forest, have a higher specificity towards the known TPE-OLD genes. To make the weights for the methods’ combination reflect the shared properties of TPE-OLD genes, they were optimized to give high ranks to the known TPE-OLD genes. This results in a ranking (Figure 2B) in which the mean percentile of the known TPE-OLD genes is 94.51% with a range from 86.21% to 97.74% (Table 2). The combined score outperforms each of the single methods both regarding the mean percentile of the known TPE-OLD genes and the lowest ranking known TPE-OLD gene (DSP).

**Table 2.** Results for known and suggested TPE-OLD genes

Gene (HGNC symbol)	Score	Rank (*n* = 18 475)	Percentile
CD9	0.8816	60	99.68%
FOXM1	0.8442	120	99.35%
TEAD4	0.7973	262	98.58%
TSPAN9	0.7867	307	98.34%
ANO2	0.7793	333	98.20%
**SORBS2**	**0.7567**	**417**	**97.74%**
**TERT**	**0.7478**	**452**	**97.55%**
CCND2	0.7407	491	97.34%
GALNT8	0.7314	551	97.02%
AKAP3	0.7193	604	96.73%
**PPP2R2C**	**0.6978**	**722**	**96.09%**
NDUFA9	0.6927	751	95.94%
**C1S**	**0.6707**	**904**	**95.11%**
**ISG15**	**0.6601**	**1038**	**94.38%**
TIGAR	0.5893	1944	89.48%
CD163L1	0.5789	2118	88.54%
**DSP**	**0.5532**	**2548**	**86.21%**

The six experimentally verified TPE-OLD genes are highlighted. These were used for developing the methods and optimizing the combined ranking. The other eleven genes have been suggested for TPE-OLD in the literature, with experimental confirmation pending. Information from these genes did not contribute to training or optimization.

At first sight, it is surprising to find both the distance score and the adjacency score to be weighted positively—an increasing distance from the telomere should diminish a gene's likelihood to be associated with TPE-OLD. And if the distance score alone is selected for the optimization, then it is indeed negatively weighted. This discrepancy is explained by the scores not being perfectly orthogonal to each other: telomeric vicinity is already partly expressed by the adjacency score and the established TPE-OLD genes all keep a distance of at least 1 Mb to the telomeres. Effectively, the distance score penalizes genes that are too close to the telomeres. The web interface can be used to assess these influences of individual methods and to identify the genes most affected by each method.

For each reference gene, the methods are applied as if the gene was not part of the reference set, i.e. using only data from the other reference genes. Therefore, the only influence of the reference genes on their own final scores and ranks stems from the final optimization on the weights to combine the individual methods. This influence was assessed by leave-one-out cross-validation and finds that the mean percentile of the reference genes drops from 94.51% to 90.12% when separately optimizing the ranking once for each reference gene based on data from the other reference genes. This indicates a good predictive power of the combined ranking considering that, during validation, predictions are made based on data from just five genes.

For an external validation, we used another set of eleven genes, that were also proposed for TPE-OLD, pending a final experimental validation: CD9, TEAD4, TIGAR, CD163L1, CCND2, GALNT8, AKAP3, TSPAN9, NDUFA9, FOXM1 and ANO2 (5). Within the combined ranking derived from the same six reference genes and without any information on the additional genes, this gene set ranks with a mean percentile of 96.29% (range 88.54–99.68%, Table 2) and significantly increased scores (*P* < 0.0001, Wilcoxon rank sum test) supporting the validity of the method.

### Graphical web interface

**Figure 3.**
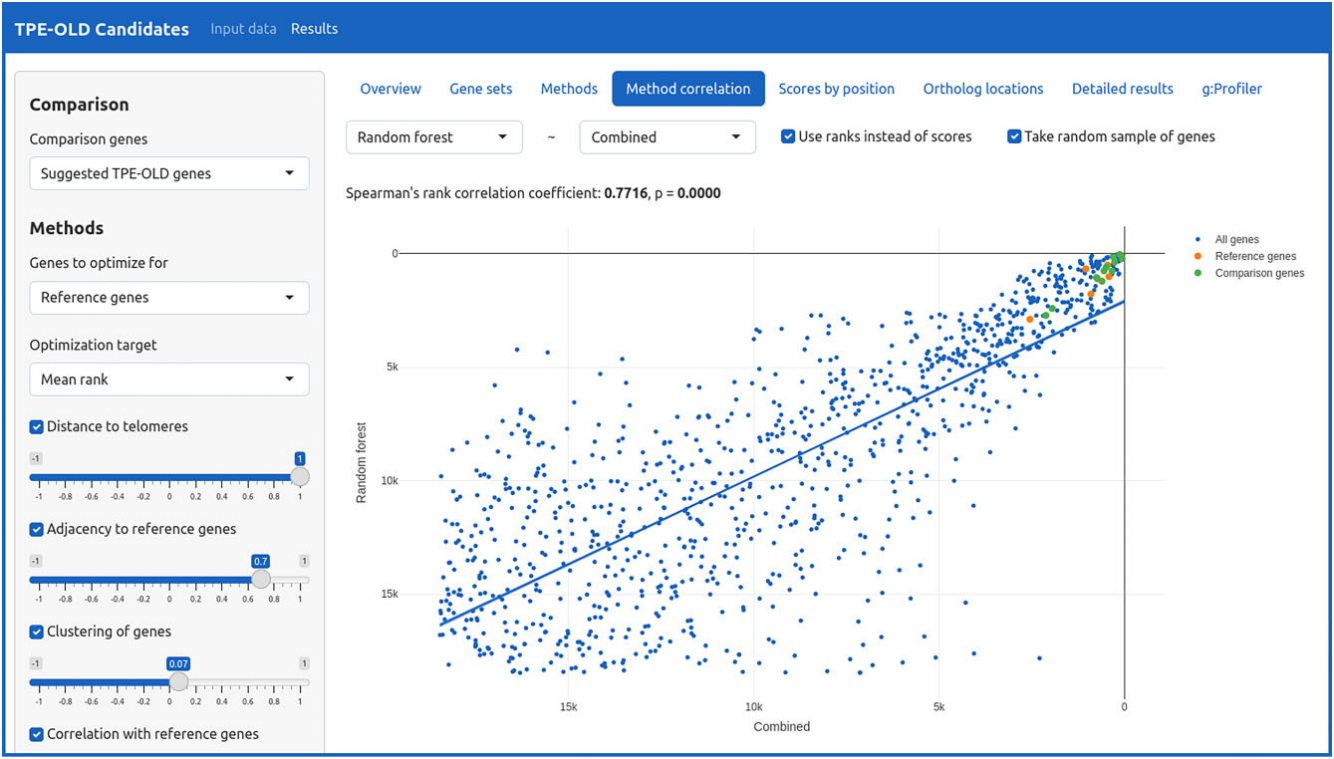
Screenshot of the interactive web interface. The interactive user interface can be used to analyze and visualize gene position data for any set of reference genes. Here, the public instance (https://tpe-old.uni-rostock.de) is shown with the predefined setup for TPE-OLD. The method parameters can be changed interactively and multiple customizable visualizations are presented. The figure shows a scatter plot comparing the ranks of the random forest method with the combined ranking. The reference genes are highlighted in orange. Users can select arbitrary genes of interest, like in this case, where eleven genes discussed in the literature for TPE-OLD are shown in green.

Visualizations of results and controls to adjust parameters are provided as an interactive graphical web interface (Figure 3), available for TPE-OLD via https://tpe-old.uni-rostock.de. Weights of each score contributing to the consensus score can be varied using sliders with immediate effect on the ranking, which enables a low-latency interactive exploration of the effects of individual weights on genes of interest (11). Among other visualizations, a presentation of scores along individual chromosomes points to local maxima of scores within larger genomic blocks.

The web interface is based on our R package implementing the method and providing an API to customize every aspect of the computations. This includes the input data (including species and reference genes), the methods to consider, the presentation and visualization of the results. Both the web interface and the R package allow applying the method beyond TPE-OLD and gene position effects in general. While all visualizations can be generated and customized using the R API, the web interface provides interactive control over their parameters.

**Figure 4.**
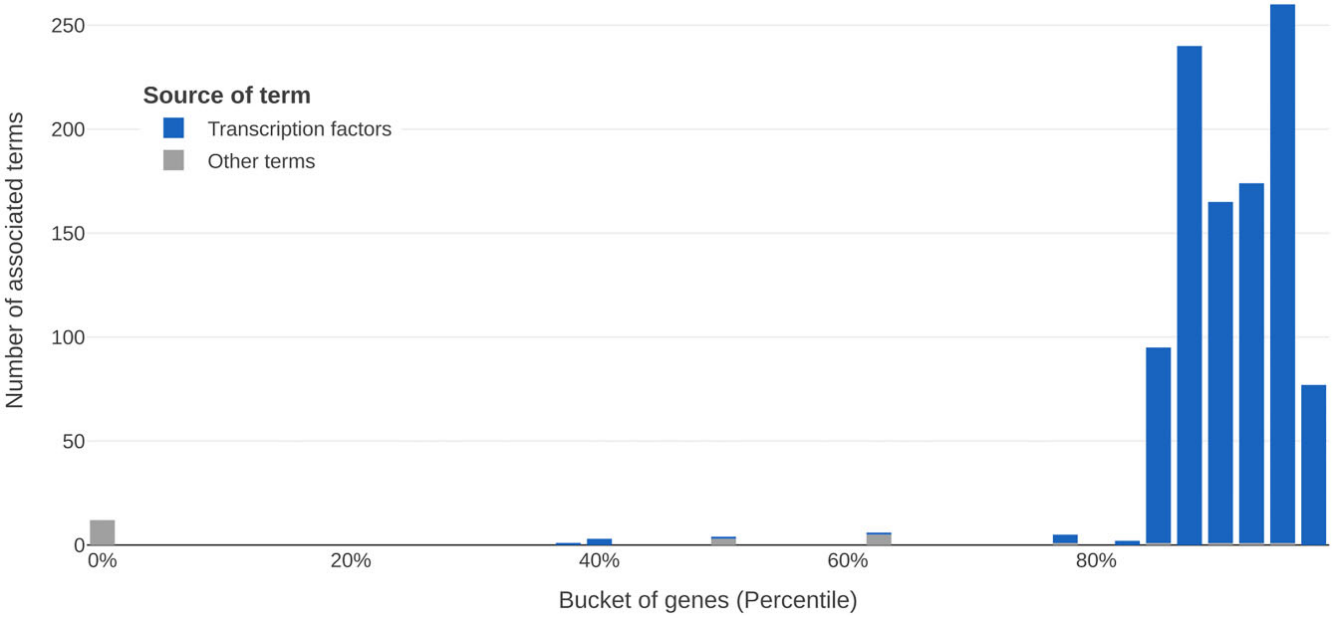
Gene set enrichment analysis along the ranking. This plot shows the results of gene set enrichment analyses along the optimized ranking for TPE-OLD. The ranking is divided into buckets of 2.5% of the analyzed genes, for a total of 462 genes per bucket. For each bucket, a gene set enrichment analysis is performed using g:Profiler. This results in a distribution of terms where the top of the ranking is notably enriched for genes with binding sites for known transcription factors indicating deep integration with regulatory mechanisms.

The web interface integrates g:Profiler (12) to directly provide gene set enrichment analyses on user-selected genes. In the final ranking, the top 5% of genes are strongly enriched for various transcription factors. This is not the case for other randomly selected, equally sized consecutive fragments of the ranking—indicating complementary regulatory mechanisms to modulate the immediate physical effects of TPE-OLD. To demonstrate this, we employ a gene set enrichment analysis along buckets of genes along the whole ranking (Figure 4). This shows a high number of associations for the top end of the ranking and zero associations for most buckets below the 80th percentile.

## Discussion

In summary, we present a computational framework for the analysis of genes, focusing on patterns in their position across species. Individual genes undergo independent scoring based on a set of straightforward heuristics. These heuristic scores can be flexibly combined to generate a composite ranking within an interactive web interface. Researchers are offered publication-ready plots, with options to constrain the analysis to individual chromosomes and tests for gene set enrichments.

Applying the method on TPE-OLD, we successfully identify well-established TPE-OLD genes with a mean percentile of 94.51% in the ranking. Additionally, the ranking confirms a set of 11 suggested TPE-OLD genes with a predicted mean percentile of 96.29%. This indicates that other top-ranking genes may also be candidates warranting further investigation in wet-lab experiments. The ranking produced using the default settings described in this publication is included in the Supplement table S1.

This work started with a cross-species cluster analysis based on Ensembl version 99 (8). With more recent versions (this analysis is based on version 110), the assemblies of genomes have been improved and additional species have been added. As expected, the exact ranking of genes is not preserved across releases of Ensembl and we find the span of scores for known TPE-OLD genes in the ranking to have shrunk and shifted towards higher scores. If our here presented approach to predict TPE-OLD affected genes gets further confirmations, with varying degrees of similarity between the genomic organization of species, one may consider the shift of the ranking of established TPE-OLD genes as additional motivation to further invest into the completion of genomic assemblies. In contrast to Jäger *et al.* (8), no distinction was made between species that may extend the length of their telomeres as an adult (replicative aging) and others (non-replicative aging); this is future work.

We can only speculate on the biomedical implications of our method. At hand is the interpretation of gene expression data for the modeling of embryonic development, cancer or aging, for which telomeric effects are known to strongly affect the clinical phenotype (7). The user may submit the list of most differentially expressed genes and is given an assessment for those genes to have elevated scores in our combined ranking. The exact interpretation beyond TPE-OLD would then depend on selecting custom reference gene sets for the analysis. Also, the bare positional conservedness independent of reference genes may be of value to integrate in the analysis of chromatin interaction data. It should be noted that the proposed scoring functions would be applicable to any other set of reference genes with distinct positional features or disease associations opening up new possibilities for studying genes based on positional data.

Applying the presented methods on TPE-OLD highlights the motivation and main use of this work: to unveil new candidate genes for research into genomic effects such as TPE-OLD which have previously been understudied. We would like to explicitly highlight the use of the web interface for exploratory research in these areas. At the same time, this means that quantitative conjectures about the positive predictive value of the ranking are difficult. We anticipate this work to positively influence decisions to investigate candidate genes for TPE-OLD, that combine a high ranking in our method, a conspicuous expression profile and associations with cellular aging and related diseases. As more TPE-OLD genes and negative findings are reported by the community allowing updates of the tool, a positive feedback loop will provide the basis for more insights into the molecular foundation of aging.

## Supplementary Material

lqae037_Supplemental_Files

## Data Availability

All input data is publicly available from Ensembl (https://www.ensembl.org). Derived data such as telomeric distances are distributed together with scripts to reproduce these within the R package. The data resulting from the application of the methods for this publication are interactively accessible via a public web interface (https://tpe-old.uni-rostock.de). A static version of the ranking for TPE-OLD as described in this paper is available at https://doi.org/10.5281/zenodo.10892254. All code used for the analyses described in this paper (https://github.com/johrpan/geposan) and the accompanying web interface (https://github.com/johrpan/geposanui) is available on GitHub under the terms of the GNU Affero General Public License. The specific versions that have been used for producing the results in this paper are available at https://doi.org/10.5281/zenodo.10892242 and https://doi.org/10.5281/zenodo.10892251.
